# Supplementary figures and images for: An aerotaxis receptor influences invasion of Agrobacterium tumefaciens into its host
Source: PeerJ. 2024 Feb 5;12:e16898. doi: 10.7717/peerj.16898 (PMC10851874; doi:10.7717/peerj.16898)

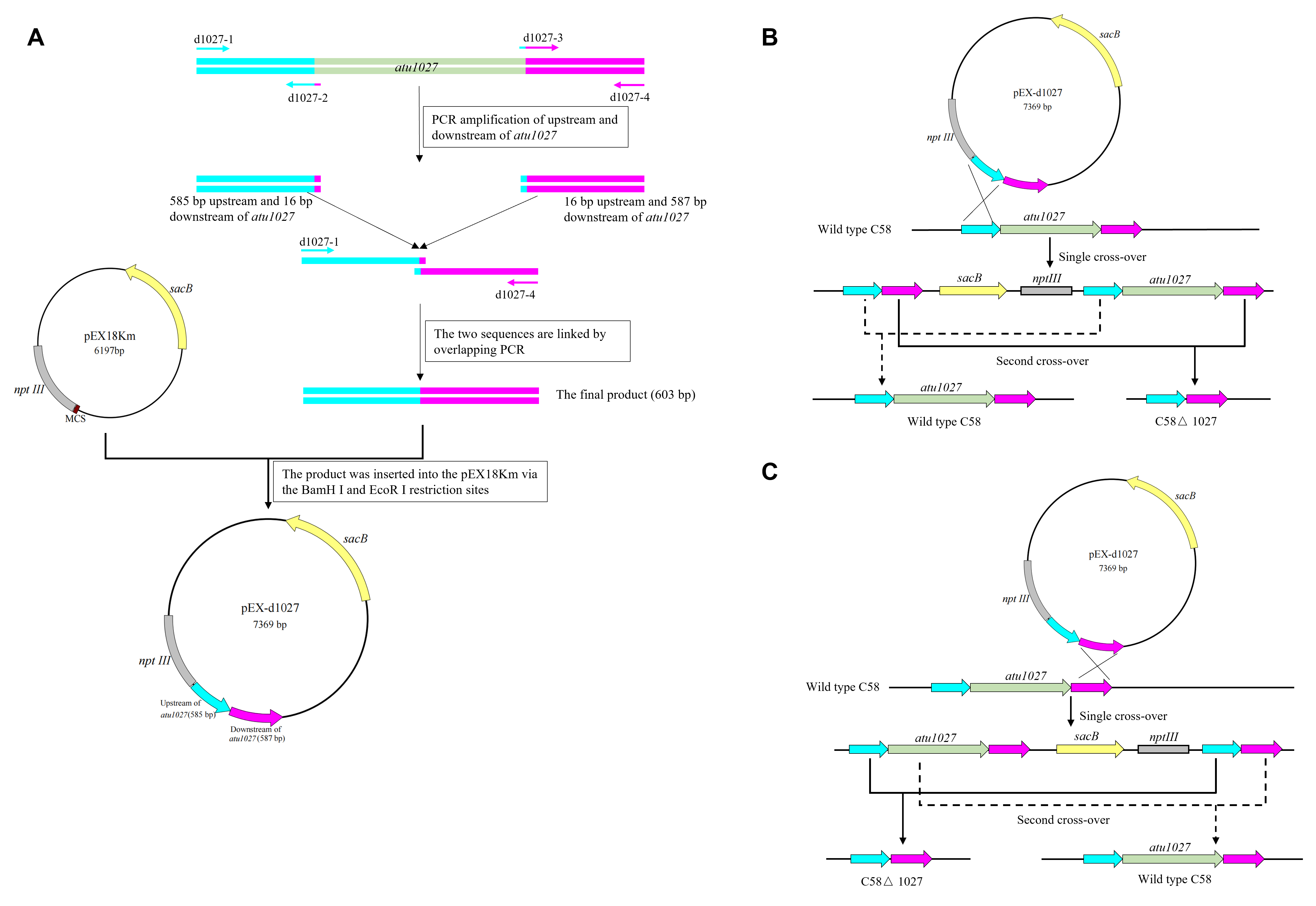

Supplement: Supplemental Information 1 — (A) the schematic depicting the process of PCR splicing through overlapping extension and the subsequent construction of the suicide plasmid pEX-d1027 is presented. Specifically, a 585 bp segment upstream and a 587 bp segment downstream of the atu1027 ORF were spliced together using overlapping PCR. These spliced segments were then inserted into the pEX18Km plasmid using the BamH I and EcoR I restriction enzymes. The resulting suicide plasmid, pEX-d1027, contained both the upstream (indicated by purple arrows) and downstream (indicated by brilliant blue arrows) sequences of the atu1027 gene. This plasmid was subsequently introduced into agrobacterial cells and integrated into the genome DNA at the targeted gene locus through homologous recombination. The sacB gene, indicated by yellow arrows, serves as a counterselectable marker gene, conferring sensitivity to sucrose. The nptIII gene, represented by gray rectangles, confers resistance to kanamycin. Colonies that have undergone single cross-over homologous recombination were screened for kanamycin resistance and sucrose sensitivity. The second cross-over homologous recombination event was identified by plating the single cross-over colonies on media containing 5% sucrose. The colonies that exhibit growth solely on MG/L media containing 5% sucrose, but not on MG/L media containing 100 µg/mL, can be utilized for identification of atu1027-deficient mutants. There are two potential strategies that can occur within cells. (A) the first strategy involves the utilization of the upstream sequence of atu1027 in a gene replacement approach. (B) the second strategy involves the utilization of the downstream sequence of atu1027 in a gene replacement approach. [file peerj-12-16898-s001.png]

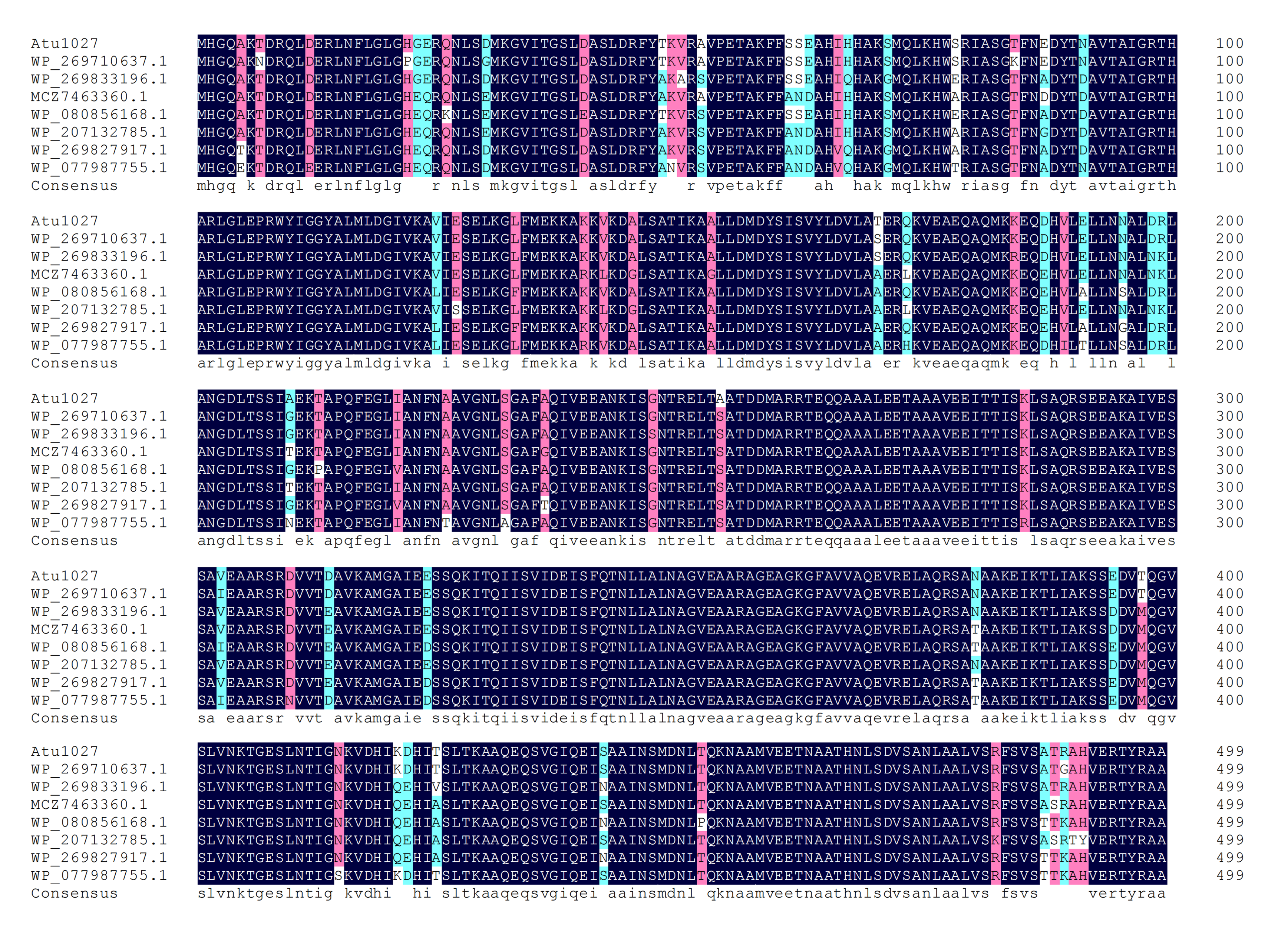

Supplement: Supplemental Information 2 — The globin domain of Atu1027 was aligned with homologous proteins including WP_269710637.1 from Agrobacterium rhizogenes, WP_269833196.1from Agrobacterium salinitolerans, MCZ7463360.1 from Agrobacterium rhizogenes, WP_080856168.1 from Agrobacterium deltaense, WP_207132785.1 from Agrobacterium burrii, WP_269827917.1 from Agrobacterium leguminum, WP_077987755.1 from Agrobacterium pusense. [file peerj-12-16898-s002.png]

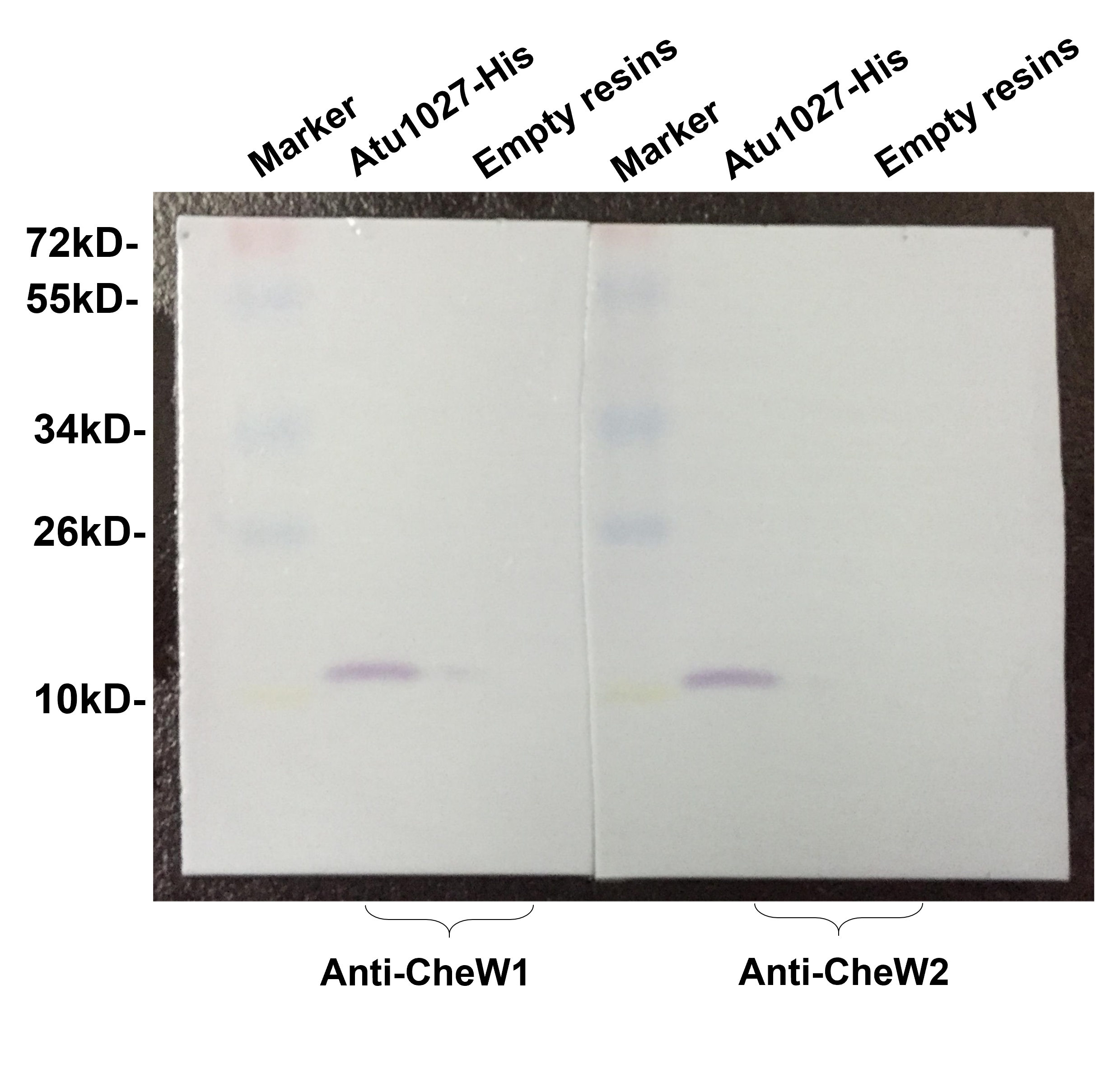

Supplement: Supplemental Information 3 [file peerj-12-16898-s003.png]

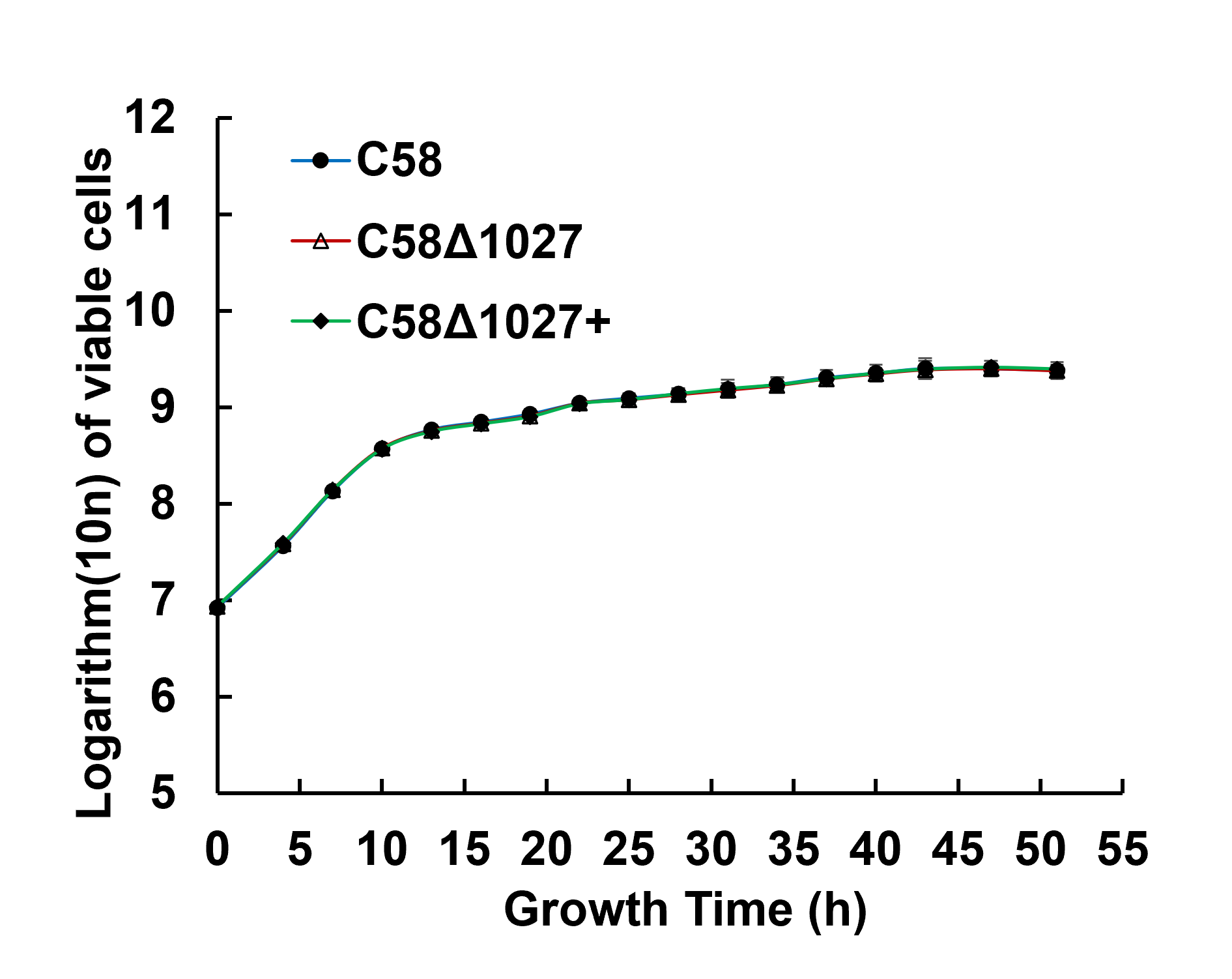

Supplement: Supplemental Information 4 — A. tumefaciens wild-type and atu1027 mutant strains were inoculated in 5 mL of MG/L medium and cultured for 12 h. Then all the tested strains were washed and re-suspended in fresh AB-sucrose medium. The cell density of different suspensions was adjusted to the same. After that, one mL of cell suspension was added to 100 mL of AB-sucrose liquid medium to determine growth curves. [file peerj-12-16898-s004.png]

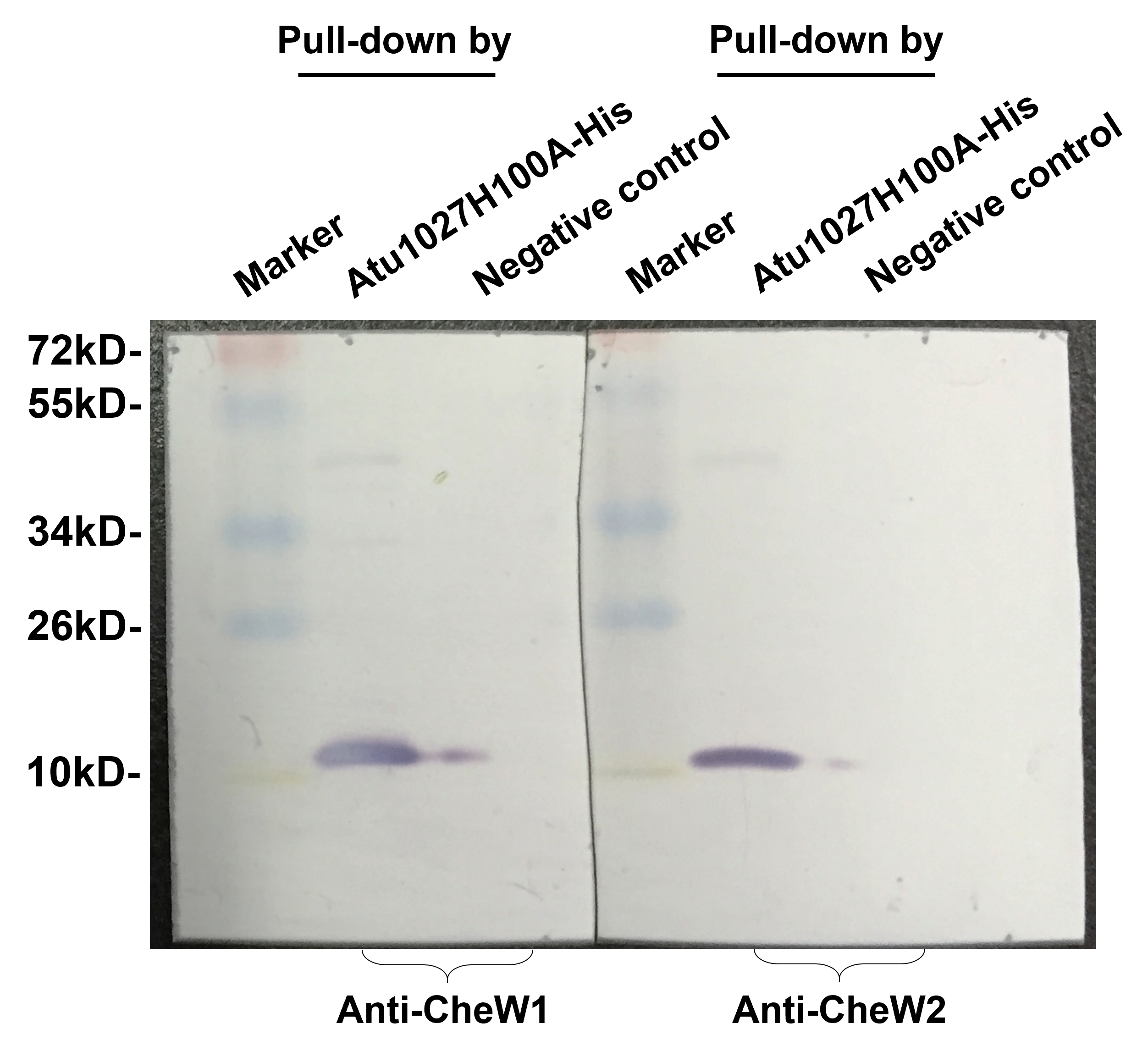

Supplement: Supplemental Information 5 [file peerj-12-16898-s005.png]

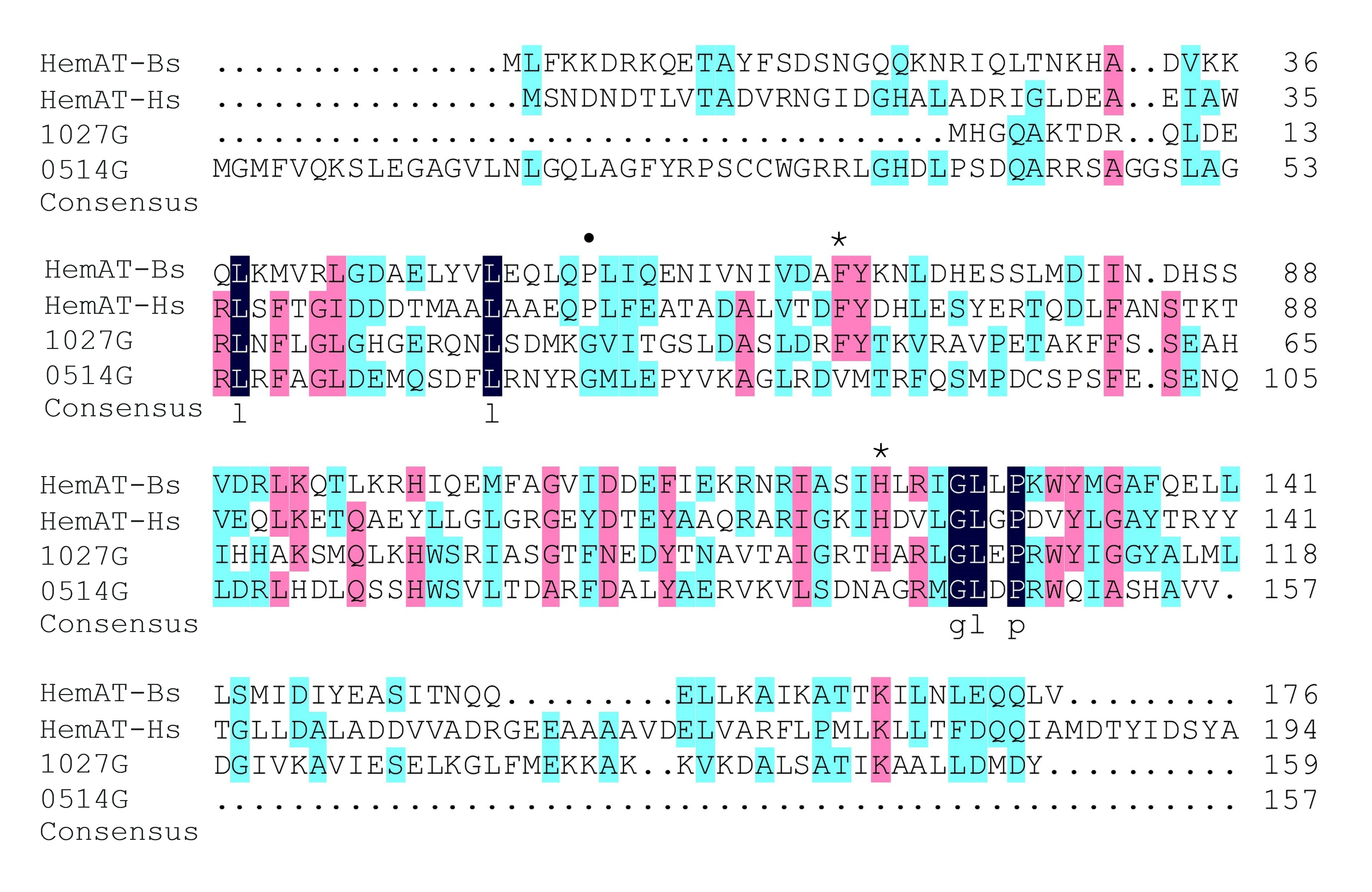

Supplement: Supplemental Information 6 — Sequences of four proteins were full aligned using the Clustal program of the DNAMAN 8. All settings used default values. In the figure on the right, the numbers indicate the position of the amino acid. Asterisks represent residues that are absolutely conserved in all globins. Dark spots represent residues that are highly conserved (Bashford, Chothia & Lesk, 1987; Vinogradov, Walz & Pohajdak, 1992; Hou et al., 2000). [file peerj-12-16898-s006.png]

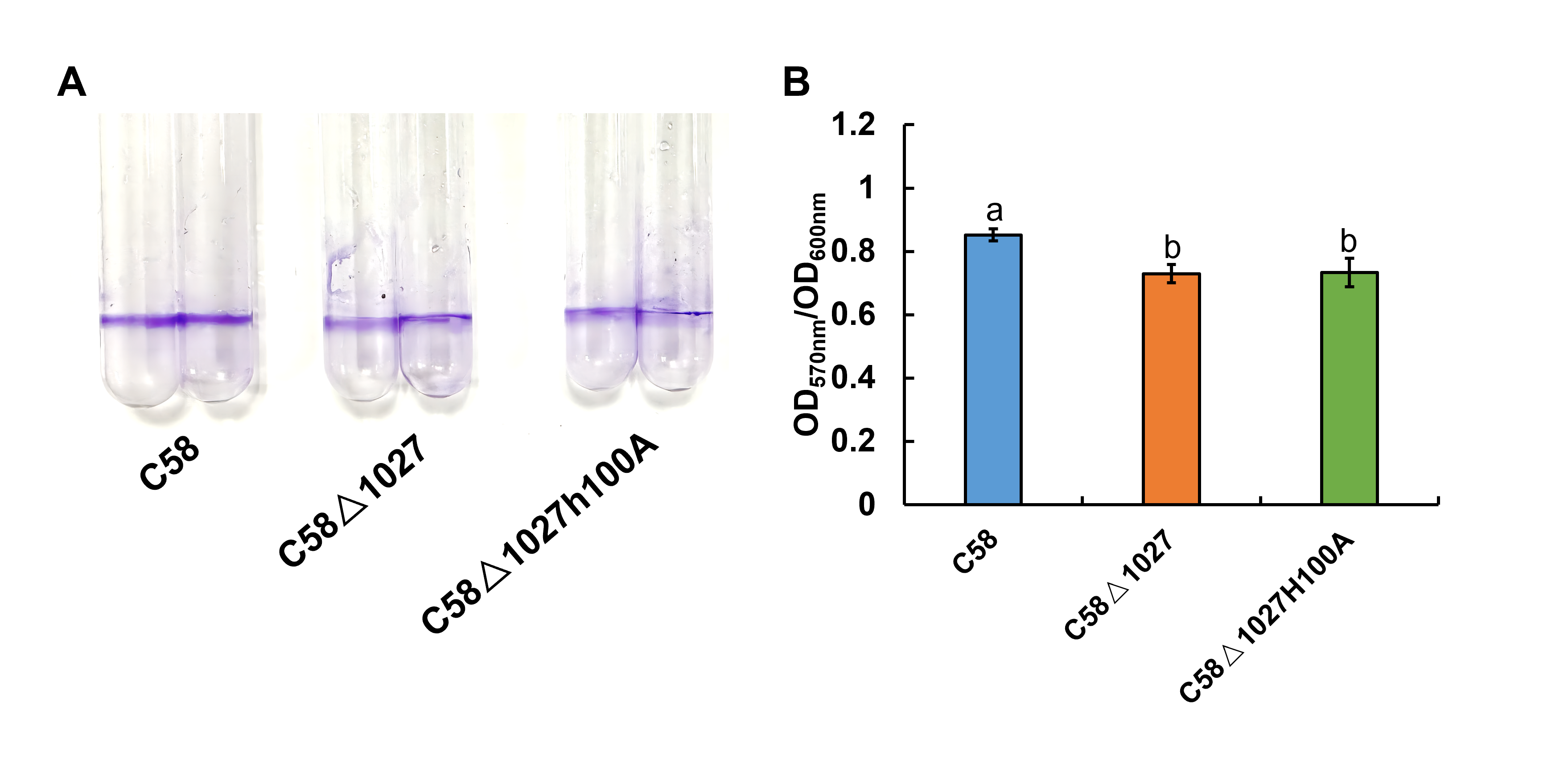

Supplement: Supplemental Information 7 — The quantification of biofilm formation in wild-type C58, C58 Δ1027, and C58Δ1027H100A was conducted using the crystal violet (CV) staining method. The OD570 value of the CV solubilized in 30% acetic acid (vol/vol) and the OD600 value of the planktonic cells were measured and computed. Distinct letters on the bar represent statistically significant differences between the various groups (P < 0.05). [file peerj-12-16898-s007.png]

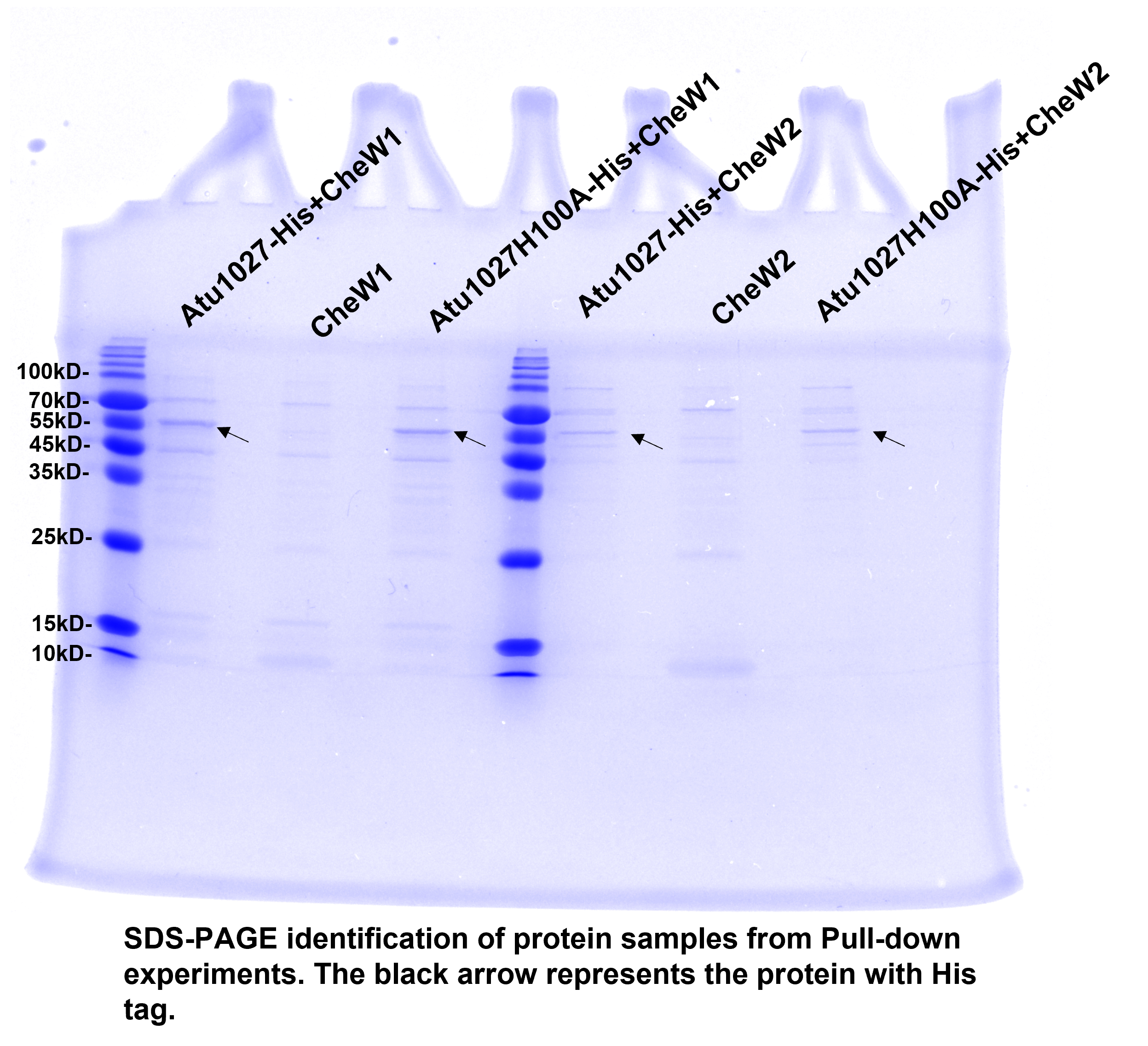

Supplement: Supplemental Information 14 [file peerj-12-16898-s014.zip › supplemental file 2/SDS-PAGE identification of protein samples from Pull-down experiments.png]

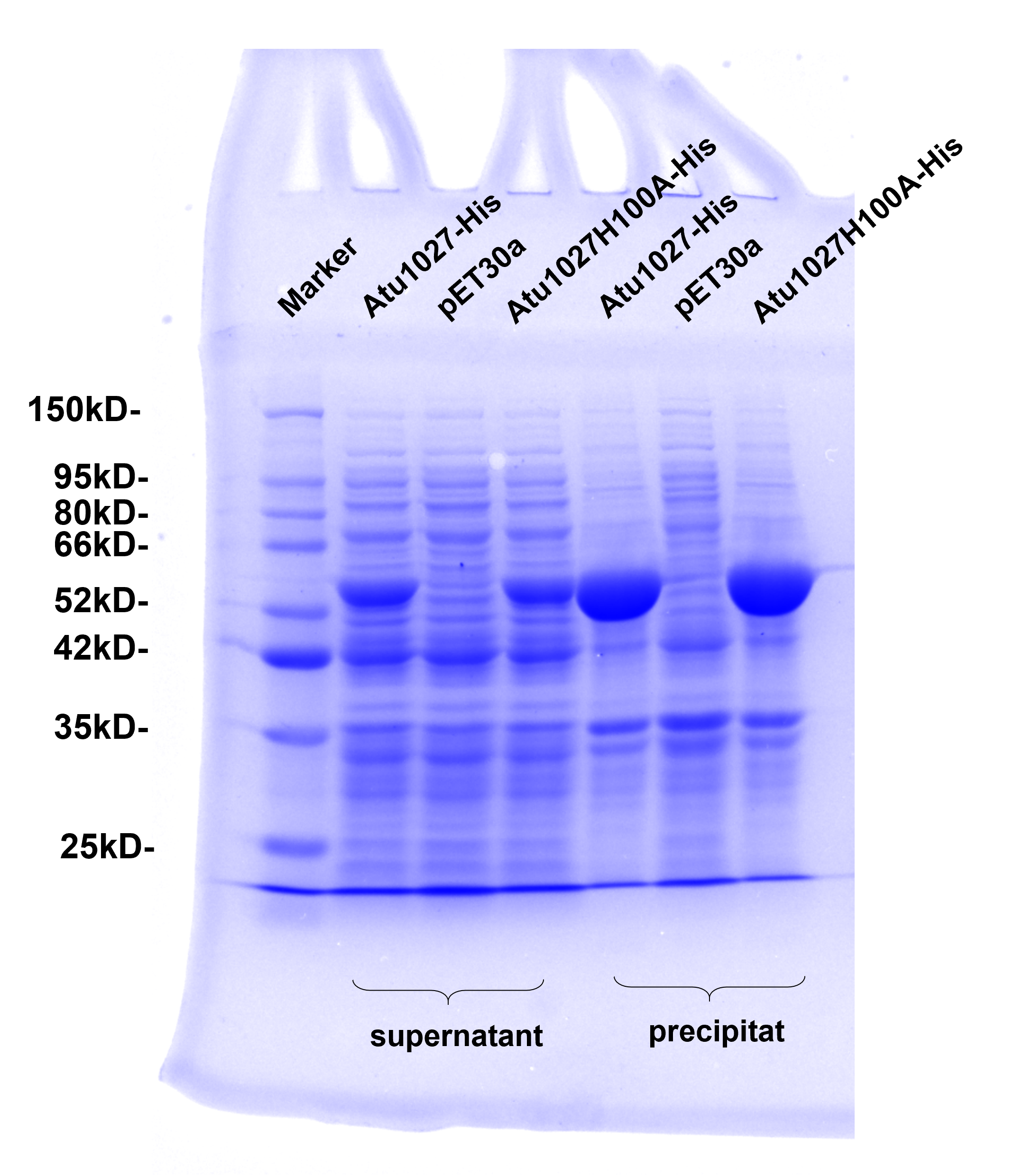

Supplement: Supplemental Information 14 [file peerj-12-16898-s014.zip › supplemental file 2/SDS-PAGE of Atu1027-His and Atu1027H100A-His.png]
