# Supplementary material for: An aerotaxis receptor influences invasion of Agrobacterium tumefaciens into its host
Source: PeerJ. 2024 Feb 5;12:e16898. doi: 10.7717/peerj.16898 (PMC10851874; doi:10.7717/peerj.16898)
Supplement: Supplemental Information 8 — Kmr = Resistant to kanamycin; Surs = Sensitive to sucrose; Apr = Resistant to ampicillin; Crr = Resistant to carbenicillin; Cmr = Resistant to chloramphenicol; Tcr = Resistant to tetracycline [file peerj-12-16898-s008.docx]

**Table S1：**

**Bacterial strain and plasmids used in this study**

| **Bacterial strains and plasmids** | **Relevant feature(s)** | **Source or reference** |
| --- | --- | --- |
| **Bacterial strains** |  |  |
| *Escherichia coli* |  |  |
| DH5α | *EndA1 hsdR17 supE44 thi-1 recA1 gyrA96 relA1 (argF-lacZYA)* U169 φ80d*lacZ*, for DNA cloning | *Huang et al., 2018* |
| BL21(DE3) | *F^–^ ompT hsdSB(rB^–^mB^–^) gal dcm (DE3),* for protein expression | *Huang et al., 2018* |
| XL1-Blue | *∆(mcrA)183 ∆(mcrCB-hsdSMR-mrr)173 endA1 supE44 thi-1 recA1 gyrA96 relA1 lac [F´ proAB lacIqZ∆M15 Tn5 (Kan^r^)],* for bacterial two-hybrid | Stratagene |
| *Agrobacterium tumefaciens* |  |  |
| C58 | Nopaline type strain; pTiC58, pAtC58 | *Guo et al., 2007* |
| C58Δ*1027* | Derivative of C58 in which *atu1027* open reading frame (orf) was deleted | This study |
| C58Δ*1027*+ | C58Δ*1027* in which *atu1027* expression was restored by pCB-1027 | This study |
| C58Δ*1027*H100A | C58Δ*1027* in which *atu1027* expression was restored by pCB-1027H100A | This study |
| C58Δ*w* | Derivative of C58 in which both *cheW_1_* and *cheW_2_* orfs were deleted, non-[chemotactic](C:/Program%20Files%20(x86)/Youdao/Dict/8.10.0.0/resultui/html/index.html#/javascript:;) strain | *Huang et al., 2018* |
| C58Δ*virA* | Derivative of C58 in which *virA* open reading frame (orf) was deleted, an avirulent strain | This study |
| **Plasmids** |  |  |
| pEX18Km | Counterselectable plasmid carrying *sacB* marker, *oriT*, Km^r^, Sur^s^ | *Huang et al., 2018* |
| pCB301 | A minim binary vector plasmid, Km^r^ | *Guo et al., 2007* |
| pET30a | Expression vector, Km^r^ | Novagen |
| pBT | The plasmid carries a p15A replication origin and confers chloramphenicol resistance. It encodes the full-length bacterial phage λcI protein under the control of the *lacUV5* promote; Cm^r^ | Stratagene |
| pTRG | The plasmid carries a ColE1 replication origin and confers tetracycline resistance. | Stratagene |
| pBT -LGF2 | Positive control plasmid, Cm^r^ | Stratagene |
| pTRG–GA111^P^ | Positive control plasmid, Tc^r^ | Stratagene |
| pEX-d1027 | pEX18Km carrying a 1,172 bp fragment, which consisted of 600 bp upstream of *atu1027* and 572 bp downstream of *atu1027*; Km^r^ | This study |
| pCB-1027 | pCB301 carrying a 2,000 bp *atu1027* expression cassette, which consisted of 500 bp promoter sequence, 1,500 bp full length *atu1027* ORF; Km^r^ | This study |
| pCB-1027H100A | A derivative of pCB-1027, in which [histidine](C:/Program%20Files%20(x86)/Youdao/Dict/8.10.0.0/resultui/html/index.html#/javascript:;) residue 100 (His100) was changed to alanine; Km^r^ | This study |
| pBT-1027 | pBT carrying a 1,500 bp full length *atu1027* ORF at EcoRI and BamHI; Cm^r^ | This study |
| pTRG-W1 | pTRG carrying a 468 bp full length *atu2075* ORF at BamHI and XhoI; Tc^r^ | This study |
| pTRG-W2 | pTRG carrying a 480 bp full length *atu2617* ORF at BamHI and XhoI; Tc^r^ | This study |
| pET30a-1027 | pET30a carrying a 1,497 bp full length *atu1027* ORF without terminator codon at NdeI and HindIII; Km^r^ | This study |
| pET30a-1027H100A | A derivative of pET30a-1027, in which histidine residue 100 (His100) was changed to alanine; Km^r^ | This study |
| pET30a-W1 | pET30a carrying 468 bp *cheW_1_* ORF at NdeI and BamHI; Km^r^ | *Huang et al., 2018* |
| pET30a-W2 | pET30a carrying 477 bp *cheW_2_* ORF at NdeI and BamHI; Km^r^ | *Huang et al., 2018* |

Km^r^ = Resistant to kanamycin; Sur^s^ = Sensitive to sucrose; Ap^r^ = Resistant to ampicillin; Cr^r^ = Resistant to carbenicillin; Cm^r^ = Resistant to chloramphenicol; Tc^r^ = Resistant to tetracycline.
