# Supplementary material for: An aerotaxis receptor influences invasion of Agrobacterium tumefaciens into its host
Source: PeerJ. 2024 Feb 5;12:e16898. doi: 10.7717/peerj.16898 (PMC10851874; doi:10.7717/peerj.16898)
Supplement: Supplemental Information 9 — a Primers in the brackets indicate that these primers can amplify more than one sequence for different purposes. b Restriction enzyme sites are highlighted with underlines. [file peerj-12-16898-s009.docx]

**Table S2：**

**Primers used in the study**

| **^a^Primers** | **^b^ Sequence** | **Length** | **Tm value** | **Purpose** |
| --- | --- | --- | --- | --- |
| d1027-1 | 5'-CGGGATCCTGGCTGGACTATAACGGCTACAAG-3' | 32 bp | 77.0 ℃ | To amplify a 601 bp fragment, which consisted of 585 bp upstream and 16 bp downstream fragment of *atu1027* |
| d1027-2 | 5'-CGCCTCTGTTGTCTTGTCTAGTCATCCTGCTCTG-3' | 34 bp | 76.3 ℃ | Over-lapping primer; to amplify a 601 bp fragment, which consisted of 585 bp upstream and 16 bp downstream fragment of *atu1027* |
| d1027-3 | 5'-GCAGGATGACTAGACAAGACAACAGAGGCGCATT-3' | 34 bp | 77.1 ℃ | Over-lapping primer; to amplify a 603 bp fragment, which consisted of 16 bp upstream and 587 bp downstream fragment of *atu1027* |
| d1027-4 | 5'-CCCAAGCTTTTCCCACCTGCCGGAATATC-3' | 29 bp | 77.0 ℃ | To amplify a 603 bp fragment, which consisted of 16 bp upstream and 587 bp downstream fragment of *atu1027* |
| cp1027-f | 5'- CGGGATCCCGACATGGATATCGAACTCGCAG-3' | 31 bp | 80.5 ℃ | To amplify a 1,979 bp *atu1027* expression cassette, which consisted of 479 bp promotor region and 1500 bp ORF of *atu1027* |
| cp1027-r | 5'-CGGAATTCCGTCAAGCAGCCCGATAGGTCC-3' | 30 bp | 79.9 ℃ | To amplify a 1,979 bp *atu1027* expression cassette, which consisted of 479 bp promotor region and 1500 bp ORF of *atu1027* |
| cp1027-H100A-2 | 5'-AGCCCCAGCCTTGCAGCGGTGCGGCCGATGGC-3' | 32 bp | 92.3 ℃ | Over-lapping primer; to amplify the first fragment (314 bp) of *atu1027* containing H100A mutation |
| cp1027-H100A-3 | 5'-GCCATCGGCCGCACCGCTGCAAGGCTGGGGCT-3' | 32 bp | 92.3 ℃ | Over-lapping primer; to amplify the second fragment (1218 bp) of *atu1027* containing H100A mutation |
| Bt1027-f | 5'-GGAATTCCATGCACGGCCAAGCAAAAAC-3' | 28 bp | 76.2 ℃ | To amplify the 1,500 bp full-length ORF of *atu1027* |
| Bt1027-r | 5'-CGGGATCCTCAAGCAGCCCGATAGGTCC-3' | 28 bp | 78.2 ℃ | To amplify the 1,500 bp full-length ORF of *atu1027* |
| BH-W1f | 5'-CGGGATCCATGTCCAACGCCATCAAGCA-3' | 28 bp | 79.9 ℃ | To amplify the 468 bp full-length ORF of *cheW_1_* |
| BH-W1r | 5'-CCCTCGAGGGCCGCTTCGCGCGCCAAC-3' | 27 bp | 87.4 ℃ | To amplify the 468 bp full-length ORF of *cheW_1_* |
| BH-W2f | 5'-CGGGATCCATGATGGCAATGATTAACTCTACCAAT-3' | 35 bp | 76.8 ℃ | To amplify the 480 bp full-length ORF of *cheW_2_* |
| BH-W2r | 5'-CCCTCGAGGGCGGCAAGATCTTCGGC-3' | 26 bp | 80.0 ℃ | To amplify the 480 bp full-length ORF of *cheW_2_* |
| dvirA-1 | 5'-CGGGATCCAAAAGCGGATAGGCGATTTGAGT-3' | 31 bp | 78.6 ℃ | To amplify a 584 bp fragment, which consisted of 566 bp upstream and 18 bp downstream fragment of *virA* |
| dvirA-2 | 5'-AAAACATCCTCAACTCCGCACTTACGTCCTCGTACCAG-3' | 38 bp | 80.1 ℃ | Over-lapping primer; to amplify a 584 bp fragment, which consisted of 566 bp upstream and 18 bp downstream fragment of *virA* |
| dvirA-3 | 5'-AGGACGTAAGTGCGGAGTTGAGGATGTTTTTCAGGAG-3' | 37 bp | 79.2 ℃ | Over-lapping primer; to amplify a 571 bp fragment, which consisted of 17 bp upstream and 554 bp downstream fragment of *virA* |
| dvirA-4 | 5'-CCCAAGCTTTGCCTTCAACATTCCCCATCTC-3' | 31 bp | 77.8 ℃ | To amplify a 571 bp fragment, which consisted of 17 bp upstream and 554 bp downstream fragment of *virA* |
| 30-1027F | 5'-GGAATTCCATATGCACGGCCAAGCAAAAACCG-3' | 32 bp | 80 ℃ | To amplify the 1,497 bp full-length *atu1027* ORF without terminator codon |
| 30-1027R | 5'-CCCAAGCTTAGCAGCCCGATAGGTCCGCT-3' | 29 bp | 78.4℃ | To amplify the 1,497 bp full-length *atu1027* ORF without terminator codon |

^a^Primers in the brackets indicate that these primers can amplify more than one sequence for different purposes.

^b^Restriction enzyme sites are highlighted with underlines.
