# Supplementary material for: An aerotaxis receptor influences invasion of Agrobacterium tumefaciens into its host
Source: PeerJ. 2024 Feb 5;12:e16898. doi: 10.7717/peerj.16898 (PMC10851874; doi:10.7717/peerj.16898)
Supplement: Supplemental Information 10 [file peerj-12-16898-s010.docx]

**Table S3:**

**Information about the sequences used in the consensus phylogenetic tree**

| **Description** | **Scientific Name** | **Query Cover** | **E value** | **Percent Identity** | **Acc. Len** | **Accession** |
| --- | --- | --- | --- | --- | --- | --- |
| globin-coupled sensor protein [*Agrobacterium fabrum*] | *Agrobacterium fabrum* | 100 | 0 | 100 | 499 | WP_010971342.1 |
| globin-coupled sensor protein [*Agrobacterium salinitolerans*] | *Agrobacterium salinitolerans* | 100% | 0 | 95.59 | 499 | [WP_269833196.1](https://www.ncbi.nlm.nih.gov/protein/WP_269833196.1?report=genbank&log$=prottop&blast_rank=1&RID=U9RT703S013) |
| globin-coupled sensor protein [*Agrobacterium deltaense*] | *Agrobacterium deltaense* | 100% | 0 | 93.79 | 499 | [WP_080856168.1](https://www.ncbi.nlm.nih.gov/protein/WP_080856168.1?report=genbank&log$=prottop&blast_rank=2&RID=U9RT703S013) |
| globin-coupled sensor protein [*Agrobacterium burrii*] | *Agrobacterium burrii* | 100% | 0 | 93.79 | 499 | [WP_207132785.1](https://www.ncbi.nlm.nih.gov/protein/WP_207132785.1?report=genbank&log$=prottop&blast_rank=3&RID=U9RT703S013) |
| globin-coupled sensor protein [*Agrobacterium pusense*] | *Agrobacterium pusense* | 100% | 0 | 92.79 | 499 | [WP_173614543.1](https://www.ncbi.nlm.nih.gov/protein/WP_173614543.1?report=genbank&log$=prottop&blast_rank=4&RID=U9RT703S013) |
| globin-coupled sensor protein [*Rhizobium oryzihabitans*] | *Rhizobium oryzihabitans* | 100% | 0 | 92.59 | 499 | [WP_082184016.1](https://www.ncbi.nlm.nih.gov/protein/WP_082184016.1?report=genbank&log$=prottop&blast_rank=5&RID=U9RT703S013) |
| globin-coupled sensor protein [*Rhizobium nepotum*] | *Rhizobium nepotum* | 100% | 0 | 90.58 | 499 | [WP_045017035.1](https://www.ncbi.nlm.nih.gov/protein/WP_045017035.1?report=genbank&log$=prottop&blast_rank=6&RID=U9RT703S013) |
| globin-coupled sensor protein [*Agrobacterium tumefaciens*] | *Agrobacterium tumefaciens* | 100% | 0 |  | 499 | WP_025592897.1 |
| globin-coupled sensor protein [*Agrobacterium cavarae*] | *Agrobacterium cavarae* | 98% | 0 | 72.86 | 496 | [WP_130978569.1](https://www.ncbi.nlm.nih.gov/protein/WP_130978569.1?report=genbank&log$=prottop&blast_rank=7&RID=U9RT703S013) |
| globin-coupled sensor protein [*Agrobacterium larrymoorei*] | *Agrobacterium larrymoorei* | 100% | 0 | 70.94 | 498 | [WP_137393538.1](https://www.ncbi.nlm.nih.gov/protein/WP_137393538.1?report=genbank&log$=prottop&blast_rank=8&RID=U9RT703S013) |
| globin-coupled sensor protein [*Agrobacterium rosae*] | *Agrobacterium rosae* | 100% | 0 | 70.14 | 498 | [WP_103587592.1](https://www.ncbi.nlm.nih.gov/protein/WP_103587592.1?report=genbank&log$=prottop&blast_rank=9&RID=U9RT703S013) |
| globin-coupled sensor protein [*Agrobacterium bohemicum*] | *Agrobacterium bohemicum* | 100% | 0 | 69.54 | 498 | [WP_067644087.1](https://www.ncbi.nlm.nih.gov/protein/WP_067644087.1?report=genbank&log$=prottop&blast_rank=10&RID=U9RT703S013) |
| globin-coupled sensor protein [*Agrobacterium vaccinii*] | *Agrobacterium vaccinii* | 100% | 0 | 69.74 | 498 | [WP_233122947.1](https://www.ncbi.nlm.nih.gov/protein/WP_233122947.1?report=genbank&log$=prottop&blast_rank=11&RID=U9RT703S013) |
| globin-coupled sensor protein [*Rhizobium album*] | *Rhizobium album* | 97% | 0 | 59.63 | 503 | [WP_109456769.1](https://www.ncbi.nlm.nih.gov/protein/WP_109456769.1?report=genbank&log$=prottop&blast_rank=12&RID=U9RT703S013) |
| globin-coupled sensor protein [*Rhizobium daejeonense*] | *Rhizobium daejeonense* | 98% | 0 | 61.43 | 504 | [WP_163904422.1](https://www.ncbi.nlm.nih.gov/protein/WP_163904422.1?report=genbank&log$=prottop&blast_rank=13&RID=U9RT703S013) |
| globin-coupled sensor protein [*Shinella lacus*] | *Shinella lacus* | 99% | 0 | 60.56 | 502 | [WP_256119832.1](https://www.ncbi.nlm.nih.gov/protein/WP_256119832.1?report=genbank&log$=prottop&blast_rank=14&RID=U9RT703S013) |
| globin-coupled sensor protein [*Shinella oryzae*] | *Shinella oryzae* | 99% | 0 | 60.36 | 502 | [WP_247217259.1](https://www.ncbi.nlm.nih.gov/protein/WP_247217259.1?report=genbank&log$=prottop&blast_rank=15&RID=U9RT703S013) |
| globin-coupled sensor protein [*Shinella curvata*] | *Shinella curvata* | 98% | 0 | 61.3 | 502 | [WP_244762521.1](https://www.ncbi.nlm.nih.gov/protein/WP_244762521.1?report=genbank&log$=prottop&blast_rank=16&RID=U9RT703S013) |
| globin-coupled sensor protein [*Pararhizobium antarcticum*] | *Pararhizobium antarcticum* | 98% | 0 | 59.39 | 503 | [WP_071830848.1](https://www.ncbi.nlm.nih.gov/protein/WP_071830848.1?report=genbank&log$=prottop&blast_rank=17&RID=U9RT703S013) |
| globin-coupled sensor protein [*Rhizobium cremeum*] | *Rhizobium cremeum* | 95% | 0 | 63.88 | 504 | [WP_244710722.1](https://www.ncbi.nlm.nih.gov/protein/WP_244710722.1?report=genbank&log$=prottop&blast_rank=18&RID=U9RT703S013) |
| globin-coupled sensor protein [*Ciceribacter lividus*] | *Ciceribacter lividus* | 100% | 0 | 58.88 | 504 | [WP_114361921.1](https://www.ncbi.nlm.nih.gov/protein/WP_114361921.1?report=genbank&log$=prottop&blast_rank=19&RID=U9RT703S013) |
| globin-coupled sensor protein [*Shinella zoogloeoides*] | *Shinella zoogloeoides* | 98% | 0 | 60.29 | 502 | [WP_160785178.1](https://www.ncbi.nlm.nih.gov/protein/WP_160785178.1?report=genbank&log$=prottop&blast_rank=20&RID=U9RT703S013) |
| globin-coupled sensor protein [*Shinella granuli*] | *Shinella granuli* | 99% | 0 | 61.17 | 502 | [WP_133033579.1](https://www.ncbi.nlm.nih.gov/protein/WP_133033579.1?report=genbank&log$=prottop&blast_rank=21&RID=U9RT703S013) |
| globin-coupled sensor protein [*Shinella sumterensis*] | *Shinella sumterensis* | 99% | 0 | 59.88 | 502 | [WP_134646029.1](https://www.ncbi.nlm.nih.gov/protein/WP_134646029.1?report=genbank&log$=prottop&blast_rank=22&RID=U9RT703S013) |
| globin-coupled sensor protein [*Ciceribacter ferrooxidans*] | *Ciceribacter ferrooxidans* | 98% | 0 | 59.27 | 504 | [WP_129331411.1](https://www.ncbi.nlm.nih.gov/protein/WP_129331411.1?report=genbank&log$=prottop&blast_rank=23&RID=U9RT703S013) |
| globin-coupled sensor protein [*Shinella pollutisoli*] | *Shinella pollutisoli* | 98% | 0 | 60.61 | 503 | [WP_257312007.1](https://www.ncbi.nlm.nih.gov/protein/WP_257312007.1?report=genbank&log$=prottop&blast_rank=24&RID=U9RT703S013) |
| globin-coupled sensor protein [*Pararhizobium arenae*] | *Pararhizobium arenae* | 100% | 0 | 57.92 | 504 | [WP_075291526.1](https://www.ncbi.nlm.nih.gov/protein/WP_075291526.1?report=genbank&log$=prottop&blast_rank=25&RID=U9RT703S013) |
| globin-coupled sensor protein [*Pararhizobium polonicum*] | *Pararhizobium polonicum* | 99% | 0 | 58.25 | 504 | [WP_068954290.1](https://www.ncbi.nlm.nih.gov/protein/WP_068954290.1?report=genbank&log$=prottop&blast_rank=27&RID=U9RT703S013) |
| globin-coupled sensor protein [*Rhizobium giardinii*] | *Rhizobium giardinii* | 96% | 0 | 60.79 | 504 | [WP_018328011.1](https://www.ncbi.nlm.nih.gov/protein/WP_018328011.1?report=genbank&log$=prottop&blast_rank=28&RID=U9RT703S013) |
| globin-coupled sensor protein [*Shinella yambaruensis*] | *Shinella yambaruensis* | 97% | 0 | 61.15 | 502 | [WP_244768206.1](https://www.ncbi.nlm.nih.gov/protein/WP_244768206.1?report=genbank&log$=prottop&blast_rank=29&RID=U9RT703S013) |
| globin-coupled sensor protein [*Ciceribacter thiooxidans*] | *Ciceribacter thiooxidans* | 97% | 0 | 58.86 | 504 | [WP_182304670.1](https://www.ncbi.nlm.nih.gov/protein/WP_182304670.1?report=genbank&log$=prottop&blast_rank=30&RID=U9RT703S013) |
| globin-coupled sensor protein [*Rhizobium terricola*] | *Rhizobium terricola* | 97% | 0 | 61.11 | 504 | [WP_169589261.1](https://www.ncbi.nlm.nih.gov/protein/WP_169589261.1?report=genbank&log$=prottop&blast_rank=31&RID=U9RT703S013) |
| globin-coupled sensor protein [*Neorhizobium vignae*] | *Neorhizobium vignae* | 95% | 0 | 57.83 | 499 | [WP_037079983.1](https://www.ncbi.nlm.nih.gov/protein/WP_037079983.1?report=genbank&log$=prottop&blast_rank=32&RID=U9RT703S013) |
| globin-coupled sensor protein [*Rhizobium wenxiniae*] | *Rhizobium wenxiniae* | 95% | 0 | 56.9 | 499 | [WP_183992945.1](https://www.ncbi.nlm.nih.gov/protein/WP_183992945.1?report=genbank&log$=prottop&blast_rank=33&RID=U9RT703S013) |
| methyl-accepting chemotaxis protein [*Rhizobium smilacinae*] | *Rhizobium smilacinae* | 95% | 0 | 56.49 | 499 | [WP_139675938.1](https://www.ncbi.nlm.nih.gov/protein/WP_139675938.1?report=genbank&log$=prottop&blast_rank=34&RID=U9RT703S013) |
| globin-coupled sensor protein [*Rhizobium terrae*] | *Rhizobium terrae* | 97% | 0 | 59.05 | 499 | [WP_117192928.1](https://www.ncbi.nlm.nih.gov/protein/WP_117192928.1?report=genbank&log$=prottop&blast_rank=35&RID=U9RT703S013) |
| globin-coupled sensor protein [*Ciceribacter selenitireducens*] | *Ciceribacter selenitireducens* | 97% | 0 | 60.91 | 504 | [WP_051438857.1](https://www.ncbi.nlm.nih.gov/protein/WP_051438857.1?report=genbank&log$=prottop&blast_rank=36&RID=U9RT703S013) |
| globin-coupled sensor protein [*Rhizobium populisoli*] | *Rhizobium populisoli* | 94% | 0 | 57.08 | 500 | [WP_219744107.1](https://www.ncbi.nlm.nih.gov/protein/WP_219744107.1?report=genbank&log$=prottop&blast_rank=37&RID=U9RT703S013) |
| globin-coupled sensor protein [*Rhizobium cellulosilyticum*] | *Rhizobium cellulosilyticum* | 95% | 0 | 55.86 | 499 | [WP_183823873.1](https://www.ncbi.nlm.nih.gov/protein/WP_183823873.1?report=genbank&log$=prottop&blast_rank=38&RID=U9RT703S013) |
| globin-coupled sensor protein [*Neorhizobium tomejilense*] | *Neorhizobium tomejilense* | 95% | 0 | 58.04 | 499 | [WP_105424708.1](https://www.ncbi.nlm.nih.gov/protein/WP_105424708.1?report=genbank&log$=prottop&blast_rank=39&RID=U9RT703S013) |
| globin-coupled sensor protein [*Neorhizobium galegae*] | *Neorhizobium galegae* | 95% | 0 | 57.41 | 499 | [WP_038592142.1](https://www.ncbi.nlm.nih.gov/protein/WP_038592142.1?report=genbank&log$=prottop&blast_rank=40&RID=U9RT703S013) |
| globin-coupled sensor protein [*Rhizobium oryzicola*] | *Rhizobium oryzicola* | 97% | 0 | 56.26 | 499 | [WP_302077290.1](https://www.ncbi.nlm.nih.gov/protein/WP_302077290.1?report=genbank&log$=prottop&blast_rank=41&RID=U9RT703S013) |
| globin-coupled sensor protein [*Ensifer sesbaniae*] | *Ensifer sesbaniae* | 95% | 0 | 60.54 | 503 | [WP_173520298.1](https://www.ncbi.nlm.nih.gov/protein/WP_173520298.1?report=genbank&log$=prottop&blast_rank=42&RID=U9RT703S013) |
| globin-coupled sensor protein [*Rhizobium deserti*] | *Rhizobium deserti* | 97% | 0 | 56.79 | 499 | [WP_133316961.1](https://www.ncbi.nlm.nih.gov/protein/WP_133316961.1?report=genbank&log$=prottop&blast_rank=43&RID=U9RT703S013) |
| globin-coupled sensor protein [*Neorhizobium petrolearium*] | *Neorhizobium petrolearium* | 97% | 0 | 57.61 | 499 | [WP_227704906.1](https://www.ncbi.nlm.nih.gov/protein/WP_227704906.1?report=genbank&log$=prottop&blast_rank=44&RID=U9RT703S013) |
| globin-coupled sensor protein [*Hoeflea phototrophica*] | *Hoeflea phototrophica* | 95% | 0 | 58.79 | 502 | [WP_007196716.1](https://www.ncbi.nlm.nih.gov/protein/WP_007196716.1?report=genbank&log$=prottop&blast_rank=45&RID=U9RT703S013) |
| globin-coupled sensor protein [*Allorhizobium sonneratiae*] | *Allorhizobium sonneratiae* | 94% | 0 | 57.17 | 504 | [WP_250939231.1](https://www.ncbi.nlm.nih.gov/protein/WP_250939231.1?report=genbank&log$=prottop&blast_rank=46&RID=U9RT703S013) |
| globin-coupled sensor protein [*Endobacterium cereale*] | *Endobacterium cereale* | 97% | 0 | 57.41 | 497 | [WP_153352453.1](https://www.ncbi.nlm.nih.gov/protein/WP_153352453.1?report=genbank&log$=prottop&blast_rank=47&RID=U9RT703S013) |
| globin-coupled sensor protein [*Allorhizobium pseudoryzae*] | *Allorhizobium pseudoryzae* | 98% | 0 | 54.76 | 501 | [WP_165222836.1](https://www.ncbi.nlm.nih.gov/protein/WP_165222836.1?report=genbank&log$=prottop&blast_rank=48&RID=U9RT703S013) |
